# New horizons in the roles and associations of COX-2 and novel natural inhibitors in cardiovascular diseases

**DOI:** 10.1186/s10020-021-00358-4

**Published:** 2021-09-30

**Authors:** Wujun Chen, Yingjie Zhong, Nuan Feng, Zhu Guo, Shuai Wang, Dongming Xing

**Affiliations:** 1 Cancer Institute, Department of Spine Surgery, The Affiliated Hospital of Qingdao University, Qingdao University, Qingdao Cancer Institute, Qingdao, 266071 Shandong China; 2 Department of Nutrition, Qingdao Women and Children’s Hospital of Qingdao University, Qingdao, 266000 Shandong China; 3 School of Medical Imaging, Radiotherapy Department of Affiliated Hospital, Weifang Medical University, Weifang, 261053 Shandong China; 4 School of Life Sciences, Tsinghua University, Beijing, 100084 China

**Keywords:** Cardiovascular, COX-2, Coxibs, Quercetin, Galangin

## Abstract

Age-related cardiovascular disease is the leading cause of death in elderly populations. Coxibs, including celecoxib, valdecoxib, etoricoxib, parecoxib, lumiracoxib, and rofecoxib, are selective cyclooxygenase-2 (COX-2) inhibitors used to treat osteoarthritis and rheumatoid arthritis. However, many coxibs have been discontinued due to adverse cardiovascular events. COX-2 contains cyclooxygenase (COX) and peroxidase (POX) sites. COX-2 inhibitors block COX activity without affecting POX activity. Recently, quercetin-like flavonoid compounds with OH groups in their B-rings have been found to serve as activators of COX-2 by binding the POX site. Galangin-like flavonol compounds serve as inhibitors of COX-2. Interestingly, nabumetone, flurbiprofen axetil, piketoprofen-amide, and nepafenac are ester prodrugs that inhibit COX-2. The combination of galangin-like flavonol compounds with these prodrug metabolites may lead to the development of novel COX-2 inhibitors. This review focuses on the most compelling evidence regarding the role and mechanism of COX-2 in cardiovascular diseases and demonstrates that quercetin-like compounds exert potential cardioprotective effects by serving as cofactors of COX-2.

## Introduction

Cardiovascular disease is the leading cause of death worldwide. Aging is a major risk factor for cardiovascular diseases (Lopez-Otin et al. 2013). By 2050, the worldwide population aged 60 years and older is expected to total 2 billion, increasing from 900 million in 2015, according to the World Health Organization (WHO). Today, 125 million people are aged 80 years or older, and by 2050, there will be almost as many people (120 million) in this age group living in China alone and 434 million people in this age group worldwide (Sendama 2020; Tyrrell and Goldstein 2020). Thus, the prevention and treatment of cardiovascular disease is a great challenge.

Cyclooxygenase-2 (COX-2) is the key rate-limiting enzyme required for the conversion of arachidonic acid (AA) to prostanoids (PGE_2_, PGD_2_, PGF_2a_, PGI_2_, and TAX_2_) Morre et al. (2020). The suppression of COX-2 is mediated by nonsteroidal anti-inflammatory drugs (NSAIDs), which are one of the most diverse classes of drugs clinically available to attenuate pain and inflammation. However, NSAIDs induce serious adverse events, including gastrointestinal (GI) and cardiovascular complications. Compared with nonselective NSAIDs, COX-2-selective drugs are known as coxibs, including rofecoxib, celecoxib, and lumiracoxib. Coxibs not only attenuate pain and inflammation but also reduce the incidence of serious GI adverse effects. However, coxibs also cause cardiovascular hazards, including atherosclerosis (coronary heart disease), hypertension, myocardial infarction, stroke, heart failure, arrhythmogenesis and sudden cardiac death (Bahmani et al. 2017; Mitchell et al. 2020). Celecoxib was removed from the market in 2004 by the Food and Drug Administration (FDA). The labels of COX-2 drugs must carry a “black box” warning to highlight the risks of serious cardiovascular events in many countries, including in the United States (US) and according to the Australian and European authorities related to the Therapeutic Goods Administration (TGA) (Arora et al. 2020; Zhu et al. 2020). Previous findings suggest that COX-2 may be a beneficial protein in the cardiovascular system. Interestingly, quercetin-like plant compounds can protect against cardiovascular diseases. Recently, quercetin-like plant compounds have been shown to act as natural cofactors of COX-2 by binding tightly to the peroxidase active site of COX-2 (Chen et al. 2020). These compounds could strongly stimulate the catalytic activity of COX-2 in vitro and in vivo at lower doses (Bai and Zhu 2008, 2010; Wang et al. 2010). We hypothesize that quercetin-like plant compounds decrease the risk of cardiovascular diseases by serving as cofactors of COX-2. In this article, we will review the most compelling evidence regarding the role of COX-2 in cardiovascular disease, and quercetin-like plant compounds exert potential cardioprotective effects by serving as cofactors of COX-2. These findings may be useful in understanding the molecular mechanism underlying the interaction between quercetin compounds and COX-2 in the cardiovascular system.

## The protective role and mechanism of COX-2 in cardiovascular disease

### COX-2 and atherosclerosis

Atherosclerosis is a major factor of coronary heart disease and is characterized by the formation of fat-laden plaques in large and medium vessels. Clinical data have shown that COX-2-selective inhibitors increase the atheroscerotic burden in patients (Bea et al. 2003; Belton et al. 2003; Burleigh et al. 2002; Burleigh et al. 2005). Global deletion of COX-2 in apoE-/- mice has been shown to accelerate atherogenesis (Yu et al. 2012). COX-2-/- mice have been shown to exhibit increased accumulation of proinflammatory factors and reduced abilities to prevent LDL oxidation and cholesterol efflux (Narasimha et al. 2007), suggesting that COX-2 protects against the development of atherosclerosis. In addition, pharmacological activation of COX-2 inhibitors also promotes the development of atherosclerosis. The COX-2 inhibitor MF-tricyclic increased the early atherosclerosis lesion area in apoE-/- mice (Rott et al. 2003). The inhibition of COX-2-derived PGE_2_ by celecoxib enhanced *P. gingivalis* LPS-induced atherosclerosis by increasing the macrophage production of TNFα (Gitlin and Loftin 2009). In another study, the COX-2-selective inhibitors celecoxib and rofecoxib also increased intermediate plaque formation in apoE-/- mice (Metzner et al. 2007). In addition, the effect of COX-2 on atherosclerosis depended on the cell type. The selective depletion of COX-2 in vascular smooth muscle cells (VSMCs) and endothelial cells (ECs) could accelerate atherosclerosis progression in low-density lipoprotein receptor (LDLR)-/-mice (Tang et al. 2014). The depletion of COX-2 in macrophages reduced atherosclerosis progression (Hui et al. 2010), suggesting that the role of COX-2 in atherosclerosis is most likely related to the cell type and atherosclerosis stage. Interestingly, COX-2 was most abundant in the thymus, brain, lung, kidney, stomach and gastrointestinal tract but not in blood vessels, as shown in COX-2^fLuc/+^ reporter mice (Kirkby 2013). COX-2 deletion accelerated atherosclerosis progression by increasing T lymphocytes in plaques (Kirkby 2014). However, COX-2 deletion did not alter vascular prostaglandin production in apoE-/- and healthy mice, suggesting that COX-2 protects against atherosclerosis independently of local vascular prostacyclin (Kirkby 2014; Kirkby et al. 2012). Taken together, these findings suggest that COX-2 can protect against atherosclerosis in vivo, but the mechanism should be further investigated.

### COX-2 and hypertension

Hypertension is a risk factor for cardiovascular disease. Evidence indicates that COX-2 plays an important role in the regulation of blood pressure (Schjerning et al. 2020). The deletion of COX-2 in C57BL6/J mice increased the blood pressure in response to both low and high salt intakes, suggesting that COX-2 activity plays a key role in blood pressure homeostasis in response to salt loading (Ricciotti et al. 2018; Staehr et al. 2013; Zhang et al. 2018). The systolic blood pressure was elevated in response to the selective inhibition (celecoxib), knockout, or mutation of COX-2 in mice with a mixed C57BL/6 × 129/Sv genetic background fed a regular chow diet (Cheng et al. 2006). In addition, a specific COX-2 pharmacological inhibitor could increase blood pressure (Zhu et al. 2020; Yao et al. 2019). Celecoxib also significantly elevated blood pressure in both normal and hypertensive rats (Huang et al. 2019). Another COX-2 inhibitor, rofecoxib, caused an increase in blood pressure dependent on PGI_2_ synthesis in normotensive Wistar-Kyoto rats (WKYs) and young spontaneously hypertensive rats (SHRs) fed a normal-salt or high-salt diet (Hocherl et al. 2002). Rofecoxib also completely prevented the hypotensive effects of the ACEi inhibitor lisinopril in SHRs (Ricciotti et al. 2018; Dubey et al. 2005). Most importantly, clinical studies suggest that hypertension was more common in patients taking COX-2 inhibitors such as celecoxib and etoricoxib, and COX inhibition may attenuate the effects of some antihypertensive therapeutics (Mitchell et al. 2020; Chan et al. 2009). Therefore, COX-2 has the ability to decrease blood pressure.

### COX-2 and myocardial ischemia–reperfusion injury

Ischemic heart disease, including acute myocardial infarction, is a major cause of death and disability worldwide. Early reperfusion is helpful for myocardial salvage but easily induces reperfusion injury, which then reduces the benefits of myocardial reperfusion. Epidemiological studies have clearly established that COX-2 alleviates myocardial ischemia–reperfusion (I/R) injury (Zhu et al. 2020; Bolli et al. 2002). Endothelial COX-2–derived PGI2 suppresses platelet aggregation. Coxibs promote thrombosis by depressing PGI2 synthesis without altering TxA2 synthesis. COX-2 promotes the recovery of left ventricular pressure after cardiac ischemia (Zhu et al. 2020). COX-2 also increases the protective effects of the late phase of ischemic preconditioning (PC) against both myocardial stunning and myocardial infarction by mediating the synthesis of PGE2 and/or PGI2. Inhibition of COX-2 activity augments myocardial cell death by obliterating the innate defensive response of the heart against I/R injury. COX-2 plays an indispensable role in protecting the heart against I/R injury (Bolli et al. 2002). COX-2 protects isolated myocytes from oxidative stress, and COX-2 inhibitors exacerbate doxorubicin-mediated myocardial injury (Adderley and Fitzgerald 1999; Dowd et al. 2001). Targeted disruption of the COX-2 gene in COX-2-knockout mice or selective deletion of COX-2 in cardiomyocytes has been shown to contribute to myocardial fibrosis and myocardial I/R injury (Dinchuk et al. 1995; Camitta et al. 2001; Papanicolaou et al. 2010). Transgene-mediated overexpression of human COX-2 protected against IR injury in mice (Inserte et al. 2009). In fact, apoptotic cell death promoted I/R injury. The inhibition of COX-2 enhanced I/R injury by promoting cell death (Dowd et al. 2001; Camitta et al. 2001). The protective role of COX-2 in myocardial I/R injury has also been identified with other molecules or drugs. Adiponectin induced COX-2 expression via a SphK-1–S1P receptor mechanism in the heart (Ikeda et al. 2008). Adiponectin protected against myocardial I/R injury by activating COX-2 and releasing PGE_2_ in cardiac cells (Li et al. 2003; Shibata et al. 2005; Minami et al. 2008). Adiponectin also promoted endothelial cell function and revascularization in ischemic muscle via a COX-2-dependent mechanism (Ohashi et al. 2009), suggesting that in the context of cardioprotection, adiponectin is closely associated with COX-2 activation. In addition, estrogen protected the heart from I/R injury via COX-2 activation and PGI_2_ synthesis (Booth et al. 2008; Xiao et al. 2001). High-density lipoprotein (HDL) has been reported to protect the heart against I/R injury by reducing cardiac TNFα levels and enhancing cardiac PGE_2_ and PGI_2_ release (Calabresi et al. 2003; Rossoni et al. 2004). HDL induced COX-2 expression and PGI_2_ release via a p38 MAPK/CRE-dependent pathway in endothelial cells (Norata et al. 2004), suggesting that HDL protected against myocardial injury through a COX-2-dependent mechanism. The beneficial effects of iNOS gene therapy on myocardial I/R injury are also associated with the upregulation of COX-2 activity (Li et al. 2003; Li et al. 2007). Peroxisome proliferator-activated receptor γ (PPARγ) agonists and recombinant human erythropoietin (rhEPO) were also effective in protecting against I/R injury in the heart by inducing COX-2 expression (Wang et al. 2012; Liu et al. 2006). Glucocorticoids protected against myocardial injury by activating COX-2 expression and lipocalin-type prostaglandin D synthase (L-PGDS)-derived PGD_2_ biosynthesis in cardiomyocytes (Tokudome et al. 2009). These findings suggest that COX-2 exerts beneficial effects on myocardial I/R injury. The beneficial effects of COX-2 on myocardial I/R injury are mainly mediated by PGI_2_, PGE_2_ and PGD_2_ through mechanisms including adenylyl cyclase antagonism, ATP-sensitive potassium channel activation, Ca^2+^ influx inhibition, and neutrophil infiltration attenuation (Bolli et al. 2002; Shinmura et al. 2002; Shinmura et al. 2000). PGE_2_ and PGI_2_ reduce myocardial I/R injury through the EP3, EP4 and IP receptors (Booth et al. 2008; Xiao et al. 2001; Xiao et al. 2004; Martin et al. 2005; Hohlfeld et al. 2000; Hishikari et al. 2009; Hirata et al. 2012). PGD_2_ and its dehydrated metabolite (15-deoxy-Δ12,14-PGJ(2)) protect the heart against I/R injury by activating Nrf2 predominantly via the FP receptor (Katsumata et al. 2014). Taken together, these findings suggest that cardiac COX-2 activity might be a promising tool for cardioprotection against myocardial I/R injury by producing PGE_2_, PGI_2_ and PGD_2_, which act through their own or other PG receptor signaling pathways.

### The possible mechanism of COX-2 inhibitor-mediated cardiotoxicity

COX-2 includes cyclooxygenase (COX) and peroxidase (POX) active sites (Chan et al. 2019; Chandel et al. 2018). AA binds to the COX active site and is converted to PGG_2_. PGG_2_ has a high binding affinity for the POX site; thus, it tightly binds to this site and is converted to PGH_2_. Finally, cell synthases and isomerases convert PGH_2_ to prostaglandins. Interestingly, COX-2 inhibitors block COX activity without affecting POX activity (Radi and Khan 2019). The phenylalanine-385 mutant of COX-2 lacks COX activity but retains POX activity, suggesting that tyrosine 385 of COX-2 is a critical residue for the initiation of COX catalysis (Yu and Funk 2007). COX-2 Y385F mice have disrupted COX activity, while POX activity is fully intact. COX-2 knockout mice have disruptions in both COX and POX activity. Interestingly, both diastolic and systolic blood pressure were elevated in COX-2 Y385F mice, COX-2 knockout mice and COX-2 inhibitor celecoxib-treated mice. These three groups of mice exhibited increased platelet consumption and thrombogenesis. The mice exhibited decreased urinary PGI_2_ metabolites, but TxA_2_ metabolites did not show overt alterations (Yu and Funk 2007; Seta et al. 2009). These results suggest that COX-2 inhibitors cause cardiotoxicity by blocking COX activity but not the POX site of COX-2.

## The possible cardioprotective effects of quercetin-like plant flavonoids as cofactors of COX-2

### Quercetin-like plant flavonoids are natural cofactors of COX-2

Recently, quercetin-like plant compounds with OH groups in their B-rings have been shown to be strong activators of the catalytic activity of COX-2 as cofactors in vitro and in vivo (Bai and Zhu 2008, 2010; Wang et al. 2010, 2018, 2019). Quercetin-like plant compounds (including quercetin, myricetin, fisetin, morin, 5,4′-dihydroxyflavone, and 7,4′-dihydroxyflavone) at very low concentrations (< 1 μM) can stimulate the formation of prostaglandins in a concentration-dependent manner (Table 1). Quercetin-like plant compounds have a high potency for activating COX-2, with an apparent EC50 value of approximately 50 nM (Bai and Zhu 2008, 2010). Specifically, quercetin compounds have the ability to bind to the COX-2 POX active site and promote COX-2 reactivation by facilitating electron transfer from compounds to heme by directly interacting with heme during a catalytic cycle (Wang et al. 2010). Most importantly, the administration of quercetin compounds strongly increased the plasma and tissue levels of several PG products in normal Sprague–Dawley rats, suggesting that quercetin-like plant compounds are naturally occurring activators of COX-2 as cofactors in vivo (Bai and Zhu 2008, 2010; Wang et al. 2010, 2018, 2019). In addition, galangin, chrysin and flavone, which have no hydroxyl groups in their B-rings, suppressed COX-2 and its mediated formation of PGs by blocking the POX site of COX-2 (Bai and Zhu 2008; Wang et al. 2018, 2019; Hyoung -Woo Bai, BT. Z. 2009; Bai et al. 2021). Curcumin also increases COX-2 expression in a time- and concentration-dependent manner (Tan et al. 2011). However, the mechanism by which curcumin acts as a cofactor of COX-2 is unclear.

**Table 1:** Chemical structures of quercetin-like natural plant compounds as cofactors of COX-2

Type	Name	Structure	References
Lead compounds	Flavonoids		(Moore 2020; Bahmani et al. 2017; Mitchell, et al. 2020; Arora et al. 2020; Duarte et al. 2002)
Activator	Quercetin		(Moore 2020; Bahmani et al. 2017; Mitchell, et al. 2020; Arora et al. 2020)
	Myricetin		(Moore 2020; Bahmani et al. 2017; Mitchell, et al. 2020; Arora et al. 2020)
	Fisetin		(Moore 2020; Bahmani et al. 2017; Mitchell, et al. 2020; Arora et al. 2020)
	Morin		(Moore 2020; Bahmani et al. 2017; Mitchell, et al. 2020; Arora et al. 2020)
	5,4′-Dihydroxyflavone		(Moore 2020; Duarte et al. 2002)
	7,4′-Dihydroxyflavone		(Moore 2020; Duarte et al. 2002)
Inhibitor	Galangin		(Chandel et al. 2018; Radi and Khan 2019; Seta et al. 2009)

Notably, quercetin compounds at higher concentrations (> 10 μM) inhibited COX-2 activity, whereas at low concentrations (10 nM), they stimulated COX-2 activity (Bai and Zhu 2008, 2010; Paoletti et al. 2009). Surprisingly, high concentrations of quercetin compounds suppressed COX-2 expression in vitro, but these compounds did not affect COX-2 expression in vivo and could even stimulate COX-2 activity (Arias et al. 2014; Pascual-Teresa et al. 2004; Nieman et al. 2007; Choi et al. 2006). Taken together, these findings suggest that quercetin-like natural plant compounds stimulate COX-2 catalytic activity by acting as cofactors of COX-2, and this effect depends on the OH structural features of their B-rings.

### Quercetin compounds exert cardioprotective effects by serving as cofactors of COX-2

Quercetin-like plant compounds have been shown to be beneficial to the cardiovascular system due to their antiatherogenic, anti-inflammatory, anticoagulative and antihypertensive effects (Deng et al. 2020; Pechanova et al. 2020; Sato and Mukai 2020). The AIN-93M diet is a flavonoid-deficient diet. The atherosclerotic plaque areas of apoE-/- mice fed the AIN-93M diet were increased by approximately 3–fourfold compared with those of C57BL/6J mice. Quercetin compounds almost completely abrogated AIN-93M-induced lesion formation in ApoE-/- mice (Loke et al. 2010). In fact, when the animals were fed a flavonoid-deficient diet, the catalytic activity of the COX-2 enzyme was very low, and the animals even died because the POX site lacked cofactors such as quercetin-like plant compounds. As mentioned above, COX-2 could reduce the atherosclerosis process. Thus, the plaque areas were increased in apoE-/-mice fed a flavonoid-deficient diet, despite the fat levels of these diets being very low (4%) (Loke et al. 2010), suggesting that quercetin-like plant flavonoids protect against the development of atherosclerosis as cofactors of COX-2.

Many studies suggest that quercetin compounds decrease blood pressure in hypertensive patients and animal models (Larson et al. 2012a). The NO and PGI_2_ pathways decrease blood pressure by relaxing blood vessels and inhibiting platelet activation. Quercetin compounds decreased the mean blood pressure by 5 mmHg in hypertensive men through a mechanism that was independent of changes in NO bioavailability (Larson et al. 2012b). Quercetin compounds also reduced the blood pressure, cardiac hypertrophy and vascular remodeling in NO-deficient rats (Duarte et al. 2002), suggesting that quercetin compounds mediate blood pressure through other mechanisms. Interestingly, quercetin compounds induced vasorelaxation through the COX-2/PGI_2_ pathway, which was not dependent on the NO pathway (Roghani et al. 2004). Consistent with these observations, we hypothesize that quercetin-like plant compounds act as cofactors of COX-2 to stimulate PGI_2_ release and then relax blood vessels to decrease blood pressure.

Quercetin compounds also play a protective role in alleviating myocardial injury (Lu 2020; Zhang et al. 2020). Quercetin compound postconditioning produced significant protective effects against myocardial I/R injury in rats by activating the PI3K/Akt signaling pathway. However, quercetin was used 5 min before reperfusion, and the heart was reperfused for 2 h (Wang et al. 2013). The PI3K/Akt signaling pathway was not activated quickly in vitro or in vivo (Liu et al. 2014). Interestingly, COX-2-mediated PGE_2_ formation reached a plateau 1 h after quercetin administration (Bai and Zhu 2010). COX-2 protects against myocardial injury by producing PGE_2_, PGI_2_ and PGD_2_, indicating that quercetin compounds induce cardioprotection via the COX-2/PG pathway in vivo. Therefore, we hypothesize that quercetin-like plant compounds may protect against myocardial I/R injury as cofactors of COX-2.

## Conclusion

**Fig. 1:**
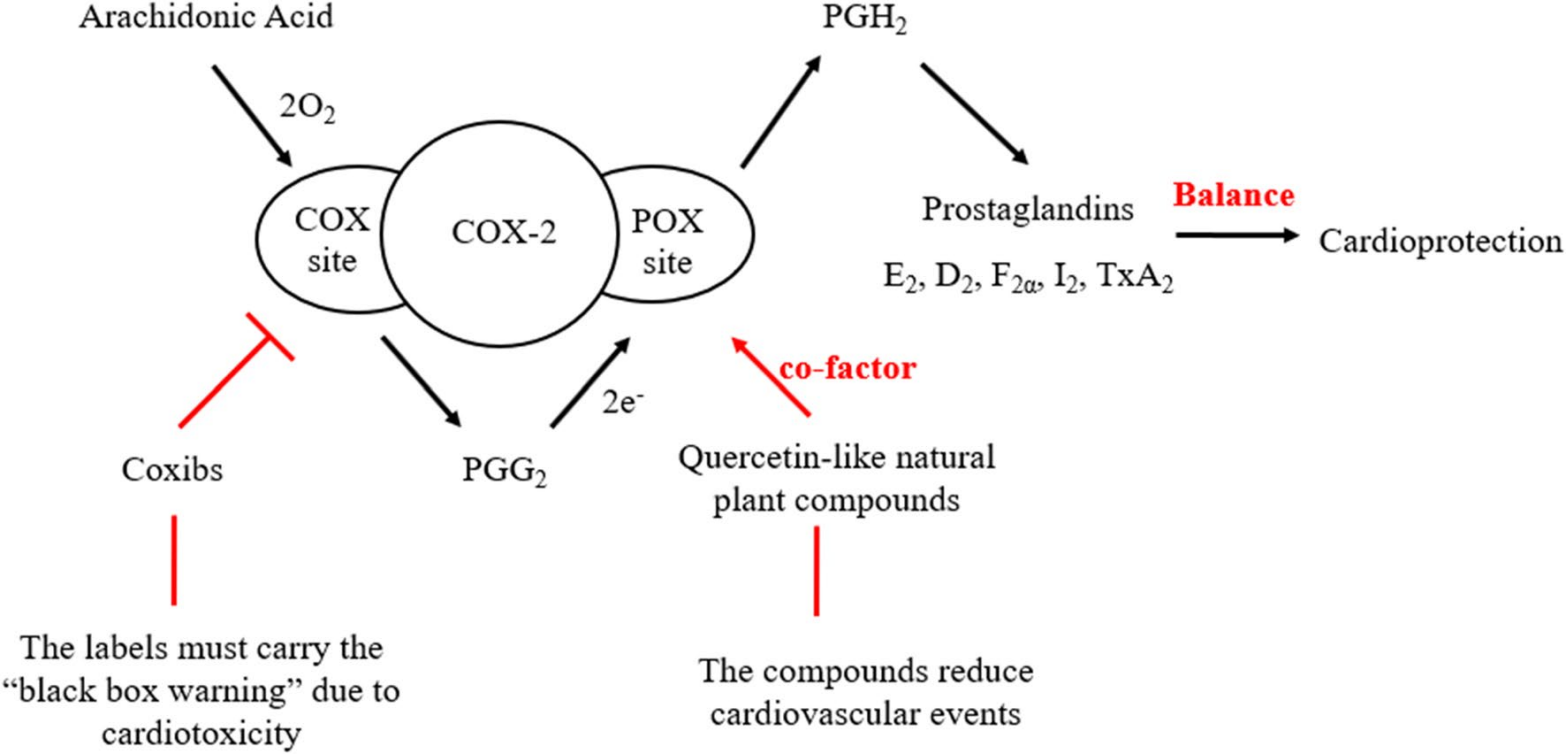
COX and POX reactions are catalyzed by coxibs and quercetin-like natural plant compounds, respectively. COX-2 catalyzes arachidonic acid conversion to PGG_2_ by the COX activity site. The POX activity site of COX-2 reduces PGG_2_ to PGH_2_. Downstream prostaglandin products are formed from PGH_2_ via different synthases. The COX activity site of COX-2 but not the POXsite is inhibited by COX-2–selective inhibitors called coxibs. The labels of coxibs must carry a “black box” warning due to adverse cardiovascular events. Quercetin-like natural plant compounds decrease the risk of cardiovascular disease and serve as activators and cofactors of COX-2 to reduce the cardiotoxicity of coxibs by binding to the POX site

Accumulating evidence has indicated that COX-2 is a beneficial protein in cardiovascular disease. Clinical studies suggest that long-term exposure to COX-2 inhibitors known as coxibs may promote the initiation of cardiovascular disease (Jeong et al. 2020; Kang et al. 2020; Liao, et al. 2020). However, clinical and rodent-based studies using coxibs have shown differential toxicity levels in the cardiovascular system, and future work is required. Of interest, quercetin-like plant compounds that are beneficial to the cardiovascular system serve as activators and cofactors of COX-2 because of the OH structural features of their B-rings (Fig. 1). Based on these observations, we suggest a new hypothesis that quercetin-like plant compounds decrease the risk of cardiovascular diseases by serving as cofactors of COX-2. We also suggest that coxibs significantly increase the risk of cardiovascular diseases in animal models fed flavonoid-deficient diets. If these hypotheses are correct, it may explain the mechanism by which coxibs are associated with a high risk of cardiovascular events in response to diets lacking certain flavonoid compounds. In addition, quercetin-like natural plant compounds usually affect multiple targets to prevent cardiovascular events. Thus, the activity of quercetin-like plant compounds as cofactors of COX-2 is just one mechanism by which they decrease the risk of cardiovascular diseases, and more research is needed to confirm this hypothesis. Galangin is present at high levels in the *Alpinia officinarum* rhizome. It is of interest that the *A. officinarum* rhizome is an herb used for conditions such as the common cold, wound swelling and pain, stomachache and diarrhea. Given that all currently used NSAIDs target the COX active sites of COX-2, galangin-like compounds that lack B-ring OH groups may serve as good lead compounds for the rational design of novel COX-2 inhibitors for clinical use as anti-inflammatory drugs by targeting the POX active sites of COX-2. Nabumetone, flurbiprofen axetil, piketoprofen-amide, and nepafenac are prodrugs that inhibit COX-2 enzymes (Sehajpal et al. 2018). The effective metabolites of these prodrugs are 6-methoxy-2-naphthyl acetic acid, flurbiprofen, ketoprofen, and amfenac (Table 2). The combination of galangin with these metabolites may lead to the development of novel COX-2 inhibitors, as ester bonds are very easily broken in vivo. We hope that more scientists will focus on the potential roles and associations of COX-2 and quercetin-like natural plant compounds in cardiovascular diseases to identify new drugs for this disease.

**Table 2:**
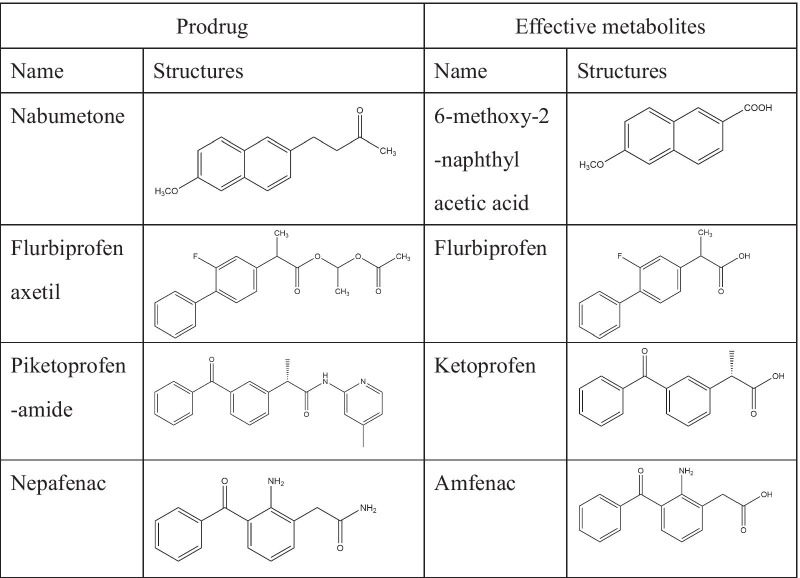
Structures of ester prodrugs obtained by inhibiting COX-2 and effective metabolites

## Data Availability

Not applicable.
